# Effects of Dietary Inclusion Level of Microwave-Dried and Press-Defatted Black Soldier Fly (*Hermetia illucens*) Larvae Meal on Carcass Traits and Meat Quality in Broilers

**DOI:** 10.3390/ani11030665

**Published:** 2021-03-02

**Authors:** Byeonghyeon Kim, Hye Ran Kim, Seul Lee, Youl-Chang Baek, Jin Young Jeong, Han Tae Bang, Sang Yun Ji, Seol Hwa Park

**Affiliations:** Animal Nutrition & Physiology Team, Rural Development Administration, National Institute of Animal Science, Wanju 55365, Korea; osorikim619@gmail.com (B.K.); ococ1004@korea.kr (H.R.K.); tabababy@korea.kr (S.L.); chang4747@korea.kr (Y.-C.B.); jeong73@korea.kr (J.Y.J.); banght80@korea.kr (H.T.B.)

**Keywords:** broiler chicken, fatty acid profile, heavy metal, *Hermetia illucens* larvae meal, meat quality

## Abstract

**Simple Summary:**

Insects are a potential source of protein for broiler diets because of the high price and limited supply of soybean. Black soldier fly (*Hermetia illucens*; HI) larvae are promising candidates as an alternative protein source to soybean meal; a microwave drying method is a convenient approach for producing HI larvae meal (HILM), as it is time- and energy-efficient. However, whether microwave-dried HILM is an appropriate protein source for producing healthy chickens for consumers has not been evaluated. Therefore, we examined the effect of 0, 25, and 50% dietary replacement of soybean meal with HILM on carcass traits, meat quality, and safety. We observed satisfactory results for the meat quality and fatty acid composition without detrimental effects on undesirable heavy metal residues in the meat. However, the carcass weight was reduced in 50% HILM-substituted diets, suggesting that a low level of HILM is beneficial. Microwave-dried HILM is a promising ingredient for broiler diets. However, further research is needed to overcome the lowering of the carcass weight in terms of utilization efficiency by improving the manufacturing process.

**Abstract:**

Limited information is available regarding the use of microwave-dried *Hermetia illucens* larvae meal (HILM) as a dietary protein source for broiler diets. Therefore, we investigated the effects of microwave-dried HILM on carcass traits, meat quality, fatty acid (FA) profiles of abdominal fat and meat, and heavy metal residues of the meat in broilers. A total of 126 male broilers were randomly assigned to three dietary treatment groups (6 replicates and 7 birds/pen): a control diet and two experimental diets in which soybean meal was replaced with 25 or 50% HILM. The broilers were slaughtered at 35 days; the carcasses were weighed, and breast and leg meats were excised from 12 birds per treatment (2 birds/pen) for meat analysis. The breast meat quality and proximate composition showed satisfactory results. For the higher HILM diet, the content of saturated FA in the abdominal fat was increased and that of polyunsaturated FA was decreased (*p* < 0.001); the FA profile of leg meat did not significantly differ between groups. The concentrations of undesirable heavy metals in the HILM and leg meat were below permissible levels. However, the carcass weight was decreased (*p* < 0.001) in the 50% HILM group. Microwave-dried HILM is a potential ingredient for broiler diets, with up to 25% substitution showing no detrimental effects on carcass traits, meat quality, FA profiles, and heavy metal residues in the meat.

## 1. Introduction

Black soldier fly (*Hermetia illucens*) larvae have been considered as a dietary source for animals because of their ability to convert organic waste to edible biomass [1,2]. Furthermore, developing black soldier fly larvae as an animal feed is a potential solution for concomitant problems related to limited food and feed given the increasing world population [3].

*Hermetia illucens* larvae meal (HILM) has been evaluated as a valuable protein source for fish and birds [4,5,6]. HILM also contains high protein and amino acid contents, which are comparable to those in soybean meal (SBM) [1]. The nutrient composition of HILM can be affected by the feeding media and rearing method of the larvae [2,7]. The fatty acid (FA) profiles of HILM can be altered by the food sources; however, saturated fatty acid (SFA) levels are typically higher than unsaturated fatty acid (UFA) levels [1]. Despite its potential advantages, toxicity can be caused by the accumulation of heavy metals in the HI larvae body via feeding of contaminated media [8,9].

HILM showed satisfactory effects on carcass traits and meat quality except for negative modulation of the FA profile in the meat of broiler quails and chickens because of the different FA composition compared with that of SBM [9,10,11]. Because the SFA content is higher than the UFA content in the HILM, the FA profile in meat was altered [5,9]. Moreover, the high content of SFA in the diet may affect the FA profile of abdominal fat because of higher fat deposition compared to polyunsaturated fatty acid (PUFA) [12]. Schiavone et al. [9] also reported that heavy metals and the arsenic level in chicken breast meat of broilers fed the HILM did not exceed the maximum allowable limits by the European Union. Moreover, HILM contains high levels of chitin, which is indigestible and can decrease protein digestibility [13]. Overall, a low inclusion level (up to 10%) of HILM appears to be suitable for broiler diets in terms of the growth performance, intestinal morphology, and FA profile of chicken meat [9,14].

Although HI larvae are a good protein source for animals in terms of their nutrient composition, appropriate processing is required to ensure nutrient availability. The nutritional quality of HILM is not only altered by substrates but also affected by the drying process, and its digestibility varies depending on the drying method used [15]. The microwave drying method has many advantages compared to conventional drying (60 °C in a drying oven) in terms of efficiency and energy use. Microwave drying transfers thermal energy from the center to the surface of products, whereas the conventional drying method involves the opposite process and is slow [16]. Microwave drying requires less space, is simpler to operate, and has lower operational costs because the walls of the apparatus do not require to be heated [16].

However, whether microwave-dried HILM can be used as a protein source for broilers is unclear. In the present study, SBM was substituted with microwave-dried HILM as a dietary protein source in broiler diets to evaluate the effects on carcass traits and breast meat quality, abdominal fat and leg meat FA composition, and heavy metal residues in chicken leg meat.

## 2. Materials and Methods

### 2.1. Birds, Diets, and Experimental Design

This study was approved by the Institutional Animal Care and Use Committee of the Rural Development Administration (No. NIAS-2020-497) and conducted at the poultry facility of the National Institute of Animal Science of South Korea. A total of 126 one-day-old male broiler chicks (Ross 308) were randomly assigned to three dietary treatments (six pens/treatment and seven birds/pen). Three experimental diets (Table 1) were formulated to meet or exceed the nutrient requirements for three phases [17]: starter (days 1–7), grower (days 7–21), and finisher (days 21–35). The control group (CON) was fed a diet based on corn and SBM; in experimental diets, SBM was substituted with 25 or 50% microwave-dried HILM (25 and 50 HILM diets, respectively). Feed and water were available *ad libitum* throughout the trial.

Cleaned and dehydrated HI larvae were dried at 70–80 °C for 30 min using a microwave drying oven; subsequently, they were press-defatted at 45–48 °C using a cold press oil machine (NF-80; Karaerler, Ankara, Turkey). The defatted larvae were pulverized to mix with other ingredients.

The chemical compositions of the experimental diets (Table 1) and the major protein sources (Table 2), excluding chitin, were analyzed using Association of Official Analytical Communities (AOAC) methods [18]. Chitin concentrations in the experimental diets and the HILM were calculated as described by Marono et al. [19].

### 2.2. Slaughtering Procedure

At the end of the study (35 days), 12 animals (2 birds per pen) from each experimental group were selected based on the average final live weight in each pen and slaughtered by cutting the carotid artery. The head, feet, and abdominal fat were removed from the plucked and eviscerated carcasses, and the carcass weight was recorded. The breast and leg meats were separated into the right and left sides and individually vacuum-sealed. The right breast meats were refrigerated at 4 °C. The abdominal fat, left breasts, and leg meats were frozen at –20 °C until further analyses.

### 2.3. Chemical Composition and Meat Quality of Breast Meat

The proximate composition of freeze-dried left breast meat samples was analyzed using AOAC methods [18]. Refrigerated right breast meats were used to measure meat quality. The breast meat color was measured at 2 different sites (CR-20; Konica Minolta, Ramsey, NJ, USA) to determine the lightness (L*), redness (a*), and yellowness (b*). The pH was measured at the same sites of the *pectoralis major* muscle using a pH meter (AM-7; Nihonseiki Kaisha Ltd., Tokyo, Japan). The meat samples were then vacuum-sealed in a plastic bag and cooked in a water bath at 80 °C until the core temperature reached 75 °C, as described by Kim et al. [20]. The samples were cooled and dried before weighing to calculate the water-holding capacity (WHC, cooking loss). Using cooked meat core samples (diameter 0.5 inches), shear force was assessed with a Warner-Bratzler shear machine (Model 4465, Instron Corp., MA, USA).

### 2.4. FA Profiles of Insect Meal, Soybean Meal, and Leg Meat

Lipid was extracted from the samples using a solvent mixture of chloroform: methanol (2:1) as described by Folch et al. [21]. FA methyl esters were separated using a gas chromatography apparatus (Star 3600; Varian Technologies, Palo Alto, CA, USA) equipped with an Omegawax 205 fused-silica bond capillary column (30 m × 0.32 mm × 0.25 µm film thickness) from the transmethylated samples using a methanolic solution of H_2_SO_4_ (4%). The oven temperature was held at 50 °C for 1 min and then raised to 200 °C at a rate of 25 °C/min. Aliquot of 2 μL of the samples were injected into the injection ports, and the temperatures of the injector and detector were 250 and 260 °C, respectively. Nitrogen was used as the carrier gas at a flow rate of 1 mL/min. The FA composition of the samples was expressed as the percentage (%) of the total detected FA methyl esters.

### 2.5. Heavy Metal Analysis

The HILM and leg meat samples (0.5 g) were mixed with nitric acid and hydrogen peroxide for microwave digestion. The resulting solutions were filtered with filter paper and transferred to acid-cleaned tubes. These solutions were diluted with deionized water. The concentrations of heavy metals in the samples were determined by inductively coupled plasma-mass spectrometry (Agilent 7700x; Agilent Technologies, Santa Clara, CA, USA).

### 2.6. Statistical Analysis

Statistical analysis of the data was performed using the GLM procedure in SAS version 9.4 (SAS, Inc., Cary, NC, USA) [22]. Differences among treatments were determined by Tukey’s multiple comparison test [22]. An individual bird was considered as an experimental unit for statistical analysis (n = 12 per treatment; two birds per pen). The results are presented as the mean ± standard error of the means. Significance and tendency were defined as *p* < 0.05 and 0.05 < *p* < 0.10, respectively.

## 3. Results

### 3.1. Chemical Composition and FA Profile of Insect Meal and SBM

The chemical compositions of HILM and SBM are summarized in Table 2. HILM contained more crude protein compared to SBM. The FA profiles differed between groups, as shown in Table 3; HILM contains the SFA more than 50%, whereas the FA profile of SBM was composed of the PUFA more than half. However, the monounsaturated fatty acid (MUFA) content was similar between the HILM and SBM groups.

### 3.2. Carcass Characteristics, Quality Parameters, and Chemical Composition of Breast Meat

As presented in Table 4, the live weight (LW) was significantly decreased (*p* < 0.001) in the 50 HILM group compared to the CON and 25 HILM groups. However, no significant difference was observed in the LW between the CON and 25 HILM groups. The carcass weight and percentage of the carcass (% LW) in the 50 HILM group were significantly lower (*p* < 0.001) than in the other groups. No significant differences were found in meat quality parameters, except for the pH, redness (a*) value, and WHC (Table 4). Dietary HILM supplementation increased (*p* < 0.05) the pH, redness, and WHC but did not significantly influence the chemical composition of the breast meat.

### 3.3. FA Profile of Abdominal Fat and Leg Meat

The effects of the experimental diets on the FA profile of abdominal fat are presented in Table 5. The proportion of SFAs, such as myristic and palmitic acid, was significantly increased, whereas PUFAs, such as linoleic, α-linolenic, and arachidonic acid, were decreased with increasing HILM levels (*p* < 0.01). The total MUFA content was also increased (*p* < 0.0001) by dietary HILM supplementation. A similar pattern was observed in the FA profile of leg meat (Table 6). However, dietary HILM supplementation did not alter the FA profile of the leg meat, contrasting the results for abdominal fat.

### 3.4. Essential Elements and Undesirable Substances in Insect Meal and Leg Meat

The concentrations of essential elements and undesirable substances in the HILM and leg meat are summarized in Table 7 and Table 8. The concentrations of undesirable substances, such as fluorine, arsenic, lead, mercury, and cadmium, in the HILM, were below the permissible limits for animal feeds according to the standards reported by the European Commission [23]. The concentrations of lead and cadmium were also under the permissible limits for chicken meats [24]. Dietary HILM increased the concentrations of selenium and zinc in the leg meat, for which permissible limits did not apply.

## 4. Discussion

Drying processes have extremely important effects on the nutritional value of food products. The microwave drying method is commonly used for food preservation and is suitable for insect processing in terms of microbial stabilization by effectively removing moisture [25]. However, the microwave drying method adversely affects the chemical composition of food by polymerizing protein particles to make them more compact with larger diameters [15]. Such chemical structural change can decrease digestibility by impairing dissolution by digestive enzymes when compared to the conventional drying methods [15,26]. Few studies have evaluated the effects of microwave-dried HILM on meat quality traits and safety. In the present study, we used a press-defatted HILM derived from larvae reared on food waste. The nutrient composition of HI larvae depends on the substrates; however, the protein content of microwave-dried HILM was superior to that of SBM [2]. HILM and SBM showed opposite FA profiles; HILM was rich in SFA, whereas SBM was rich in PUFA. Thus, we predicted that dietary microwave-dried HILM would affect carcass traits and the FA composition of the abdominal fat and meat. 

In the present study, increasing the level of substitution of SBM with HILM in the broiler diet negatively affected carcass traits. The decreased live and carcass weights of broilers in the 50 HILM group could be attributed to the chitin in the exoskeleton of HI larvae [13]. It was reported that chitin can negatively affect protein digestibility [19]. Acid detergent fiber (ADF) fractions were analyzed to estimate the chitin contents in the HILM and the diets. The amount of the chitin in the HILM used as feed in the present study was 5.70%, calculated as follows: chitin (%) = ash free ADF (%) − ADF-linked protein (%), according to Marono et al. [19]. In the present trial, average daily feed intakes in the CON, 25 HILM, and 50 HILM groups were 70.30, 70.69, and 62.07 g/d, respectively. Therefore, the 25 and 50 HILM groups ingested approximately 0.28 and 0.50 g/d of chitin, respectively, considering the inclusion levels of HILM in the diets. According to Bovera et al. [4], laying hens (sixteen weeks old) that ingested approximately 0.47 g/d of chitin had lower protein digestibility than those fed on SBM-based diets, and it linearly decreased with an increase in inclusion level of insect meal in the diet. Chitin, which cannot be digested, and the microwave drying process of HILM may explain these lowered carcass traits [4,13]. However, the 25 HILM group showed satisfactory results for the carcass weight and rate compared to the 50 HILM group. This result agrees with that of a previous study showing that the carcass weight was linearly decreased with the inclusion levels (5, 10, and 15%) of HILM and drastically decreased by high inclusion level (15%) of HILM [9]. Therefore, including a low level of HILM in the broiler diet may maintain the carcass traits.

In general, the pH of meat is correlated with the WHC, and changes in the pH of breast meat in broilers fed HILM were related to the carcass weight. Therefore, the increased WHC was influenced by the meat pH [27]. Additionally, the pH of meat was negatively correlated with carcass weight, and the decreased carcass weight of broilers fed HILM affected the pH of breast meat [28,29]. Although the pH of breast meat was increased by dietary HILM, the pH and L* values of breast meat were in normal ranges [30,31]. Bovera et al. [32] also reported that the use of *Tenebrio molitor* larvae meal in a broiler diet increased the breast meat pH, whereas inclusion of HILM did not affect the pH regardless of the carcass weight loss [9]. Additionally, the substitution of HILM in a broiler quail diet decreased the pH in breast meat without negatively affecting the carcass weight and dressing percentage [10]. They suggested that the difference in pH occurred because of differences in the muscle glycogen content. These conflicting results for the pH of breast meat in broilers fed insect meal should be interpreted with caution. 

According to the meat quality parameters, HILM inclusion had a satisfactory effect on the chicken breast meat. The increase in the pH and WHC did not affect cooking loss or shear force. This result indicates that chicken breast meat from broilers fed HILM can contribute to consumer acceptance of the meat [29]. However, when HILM was included in the broiler diets, a noticeable increase in redness was observed, which agrees with a previous study [9]. They also showed some conflicting results; yellowness was decreased by dietary HILM. In contrast, redness was decreased and yellowness was not affected in the breast meat of broiler quails [10]. The reason for the higher level of redness in breast meat in the 25 and 50 HILM groups compared with those in the CON group are unclear, although different species and breeds may affect the meat quality [33]. Additionally, a pigment derived from HILM and HI larvae oil may have affected the meat color and egg yolk color [9,11].

As expected, the FA profile in abdominal fat changed in line with the pattern of that in HILM, which contained higher levels of SFAs and lower levels of PUFAs than SBM. However, dietary HILM showed weaker effects on the FA profile of leg meat. Similarly, previous studies showed that the FA profile of breast meat from broiler chicken and quail was affected by the high SFA content in HILM [9,10]. In the present study, the unchanged FA composition in leg meat was attributed to the relatively lower SFA content in microwave-dried HILM when compared to the levels in previous studies. Moreover, the FA composition of fat tissues (e.g., abdominal and subcutaneous) is more representative of the dietary FA composition than that in muscle tissue such as breast and leg muscles of broiler chickens [34]. Particularly, it was reported that broilers fed PUFA had lower abdominal fat levels, with more fat deposited in the muscle because of the different roles of SFA and PUFA in the body tissues [12]. This explains the significant difference in the FA composition of abdominal fat but not in leg meat. In a previous study, it was reported that the FA profile of abdominal fat was not changed in broilers fed *Tenebrio molitor* larvae meal [35]. The different FA concentrations of the *Tenebrio molitor* and HI can be attributed to the conflicting result; UFA concentrations are higher in mealworms than HI larvae [36].

The safety of chicken leg meat from broilers fed HILM must be verified to determine its heavy metal concentrations, ensuring that healthy chickens were provided to consumers and to improve consumer perception. Heavy metals accumulated in all development stages (e.g., larvae, prepupae, and adults); heavy metal concentrations in the larvae were increased with metal concentrations in the food [37]. The concentrations of hazardous heavy metals such as fluorine, arsenic, lead, mercury, and cadmium in HILM were below the European Union limits [23]. Moniello et al. [38] also reported concentrations of the toxic elements in insect meal and experimental diets in which SBM was replaced with insect meal (inclusion level in the diets: 7.3 and 14.6%, respectively). The concentrations of the toxic elements in the microwave-dried HILM were comparable or lower than those in a previous study [39], demonstrating that the concentrations of the toxic elements were negligible [39]. Our results also suggest that the microwave-dried HILM is suitable as an animal feed ingredient with regard to safety. Moreover, the concentrations of lead and cadmium in the leg meat did not exceed allowable levels [24]. In this study, the contents of zinc and sulfur in leg meat were increased by dietary HILM but the level of the zinc was in a normal range and not defined by the European Union [39]; the allowable level of sulfur is also not legislated [24]. In general, sulfur is provided by sulfur-containing amino acids (e.g., methionine and cysteine) in humans, and most people may not ingest sufficient amounts of sulfur [40]. These results suggest that HI larvae fed food waste was not contaminated, with no negative effects on chicken meat from broilers fed HILM.

## 5. Conclusions

We demonstrated the effects of microwave-dried HILM as a potential feed ingredient in broiler diets. The difference in the FA profiles of abdominal fat between dietary treatments was ascribed to the different FA profiles of HILM and SBM. The FA profile showed lower differences in chicken meat than in abdominal fat, and the breast meat showed good quality. Dietary HILM did not negatively affect the heavy metal contents in the chicken leg meat. Our findings suggest that SBM can be substituted at less than 25% with HILM in the broiler diets in terms of carcass weight. Further studies are needed to examine the utilization efficiency and improve the manufacturing process of HILM.

## Figures and Tables

**Table 1:** Ingredients and chemical composition of the experimental diets (as-fed basis).

Item	Starter			Grower			Finisher		
	CON	25 HILM	50 HILM	CON	25 HILM	50 HILM	CON	25 HILM	50 HILM
Ingredients, %									
Corn	52.69	57.21	63.15	56.03	60.89	65.72	59.32	63.10	67.86
Soybean meal, 45.8%	30.00	21.50	12.00	28.00	19.00	11.00	26.00	18.50	11.00
HILM	-	7.50	15.00	-	7.00	14.00	-	6.50	13.00
Corn gluten meal	8.00	7.00	6.00	7.00	6.00	5.00	6.00	5.00	4.00
Soybean oil	4.50	3.00	1.00	4.50	3.50	1.50	4.50	3.50	1.50
Dicalcium phosphate	1.95	1.80	1.60	1.80	1.70	1.60	1.60	1.56	1.54
Limestone	1.50	0.70	-	1.40	0.70	-	1.40	0.70	-
L-lysine	0.45	0.41	0.39	0.36	0.32	0.30	0.30	0.28	0.26
DL-methionine	0.16	0.13	0.11	0.16	0.14	0.13	0.13	0.11	0.09
Salt	0.25	0.25	0.25	0.25	0.25	0.25	0.25	0.25	0.25
Vitamin-mineral premix ^(1)^	0.50	0.50	0.50	0.50	0.50	0.50	0.50	0.50	0.50
Calculated composition									
ME, kcal/kg	3119	3119	3119	3149	3149	3149	3177	3177	3177
Lysine, %	1.47	1.47	1.47	1.33	1.33	1.33	1.21	1.21	1.21
Methionine, %	0.55	0.55	0.55	0.53	0.53	0.53	0.48	0.48	0.48
Calcium, %	0.97	0.97	0.97	0.91	0.91	0.91	0.85	0.85	0.85
Total phosphorus, %	0.79	0.79	0.79	0.75	0.75	0.75	0.70	0.70	0.70
Chitin, %	-	0.43	0.86	-	0.40	0.80	-	0.37	0.74
Analyzed composition, %									
Crude protein	23.23	23.67	23.60	21.96	21.76	22.08	19.53	19.97	19.80
NDF	8.67	10.03	11.08	7.90	8.76	9.99	7.35	7.47	7.90
ADF	3.36	4.03	4.38	3.73	3.67	4.02	3.10	3.59	3.74
ADF-linked protein	0.42	0.91	1.02	0.68	0.82	1.00	0.48	0.79	1.09
Ash	8.34	7.50	8.20	6.90	7.46	7.18	7.46	7.43	7.57

CON, control diet; 25 and 50 HILM, HILM groups in which the soybean meal was replaced with 25 and 50% HILM (*Hermetia illucens* larvae meal), respectively; ME, metabolizable energy; NDF, neutral detergent fiber; ADF, acid detergent fiber; ^(1)^ Supplied per kilogram of diet: vitamin A 1,600,000 IU; vitamin D_3_ 300,000 IU; vitamin E 800 IU; vitamin K_3_ 132 mg; vitamin B1 97 mg; vitamin B2 500 mg; vitamin B6 200 mg; vitamin B12 1.2 mg; nicotinic acid 2000 mg; pantothenic acid 800 mg; folic acid 60 mg; choline chloride 35,000 mg; Mn 12,000 mg; Zn 9000 mg; Fe 4000 mg; Cu 500 mg; I 250 mg; Co 100 mg; Se 50 mg.

**Table 2:** Chemical composition (%) of *Hermetia illucens* larvae meal (HILM) and soybean meal.

Item	HILM	Soybean Meal
Dry matter	98.53	87.95
Crude protein	61.24	45.76
Ether extract	6.16	2.23
NDF	20.34	10.14
ADF	11.24	9.50
ADF-linked protein	5.54	3.46
Ash	14.98	5.52
Chitin ^(1)^	5.70	-

NDF, neutral detergent fiber; ADF, acid detergent fiber; ^(1)^ Calculated data.

**Table 3:** Fatty acid profile (% of total fatty acid methyl esters) of *Hermetia illucens* larvae meal (HILM) and soybean meal.

Item	HILM	Soybean Meal
Fatty acids		
C10:0	1.62	0.02
C12:0 (Lauric)	29.69	0.15
C14:0 (Myristic)	4.39	0.46
C16:0 (Palmitic)	15.85	15.87
C17:0	0.23	0.18
C18:0 (Stearic)	3.37	6.21
Total SFA	55.15	22.89
C14:1	0.10	0.06
C16:1 *n*-7 (Palmitoleic)	2.67	0.69
C17:1	0.23	0.09
C18:1 *n*-9 (Oleic)	19.67	22.39
C20:1 *n*-9 (Eiocosenoic)	0.20	0.21
Total MUFA	22.87	23.44
C18:2 *n*-6 (Linoleic)	15.74	46.19
C18:3 *n*-3 (α-Linolenic)	2.58	5.35
C20:2 *n*-6	0.20	0.19
C20:3 *n*-3	0.03	0.03
C20:4 *n*-6 (Arachidonic)	0.28	0.14
Total PUFA	18.80	51.87
UFA/SFA	0.76	3.29
*n*-6	16.22	46.52
*n*-3	2.61	5.38
*n*-6/*n*-3	6.21	8.65

SFA, saturated fatty acid; MUFA, monounsaturated fatty acid; PUFA, polyunsaturated fatty acid; UFA, unsaturated fatty acid.

**Table 4:** Effect of dietary *Hermetia illucens* larvae meal (HILM) inclusion level on the carcass and breast meat traits of broiler chickens.

Item	Dietary Treatment			SEM	*p*-Value
	CON	25 HILM	50 HILM		
Live weight (LW), kg	1.80 ^a^	1.76 ^a^	1.49 ^b^	0.04	0.0004
Carcass weight, kg	1.27 ^a^	1.25 ^a^	1.03 ^b^	0.03	0.0003
Carcass weight, % LW	70.67 ^a^	71.16 ^a^	68.91 ^b^	0.53	0.0003
pH	5.75 ^b^	5.77 ^ab^	5.87 ^a^	0.03	0.0409
Color					
Lightness, L*	54.79	55.52	55.21	1.03	0.8815
Redness, a*	3.52 ^b^	5.16 ^a^	5.13 ^a^	0.34	0.0023
Yellowness, b*	8.11	8.87	8.72	0.78	0.7661
Cooking loss, %	23.71	23.15	22.60	0.74	0.6207
WHC, %	60.39 ^ab^	60.05 ^b^	62.24 ^a^	0.57	0.0422
Shear force, kg/0.5 inch^2^	2.48	2.42	2.34	0.19	0.9017
Moisture, %	75.75	75.85	76.27	0.25	0.3796
Protein, %	22.43	22.27	22.21	0.24	0.8225
Lipid, %	0.95	1.16	0.87	0.11	0.2024
Ash, %	1.09	1.08	1.02	0.02	0.1335

CON, control diet; 25 and 50 HILM, HILM groups in which the soybean meal was replaced with 25 and 50% HILM, respectively; SEM, standard error of the means; WHC, water-holding capacity; ^a,b^ Values with different superscripts in the same row are significantly different (*p* < 0.05).

**Table 5:** Effect of dietary *Hermetia illucens* larvae meal (HILM) inclusion level on the fatty acid profile in abdominal fat of broiler chickens.

Item	Dietary Treatment			SEM	*p*-Value
	CON	25 HILM	50 HILM		
Fatty acids					
C14:0 (Myristic)	0.43 ^c^	0.68 ^b^	0.85 ^a^	0.03	<0.0001
C16:0 (Palmitic)	20.84 ^c^	23.25 ^b^	25.44 ^a^	0.53	<0.0001
C18:0 (Stearic)	7.31	6.79	6.53	0.25	0.1133
Total SFA	28.57 ^c^	30.72 ^b^	32.83 ^a^	0.54	0.0002
C16:1 *n*-7 (Palmitoleic)	4.12 ^c^	5.46 ^b^	6.88 ^a^	0.35	0.0002
C18:1 *n*-9 (Oleic)	38.57 ^b^	39.37 ^b^	42.44 ^a^	0.52	0.0002
C20:1 *n*-9 (Eicosenoic)	0.38	0.36	0.38	0.01	0.2764
Total MUFA	43.06 ^b^	45.19 ^b^	49.70 ^a^	0.76	<0.0001
C18:2 *n*-6 (Linoleic)	26.28 ^a^	22.28 ^b^	16.27 ^c^	0.98	<0.0001
C18:3 *n*-6 (γ-Linolenic)	0.21	0.20	0.17	0.01	0.1820
C18:3 *n*-3 (α-Linolenic)	1.61 ^a^	1.43 ^a^	0.89 ^b^	0.07	<0.0001
C20:4 *n*-6 (Arachidonic)	0.26 ^a^	0.18 ^ab^	0.14 ^b^	0.02	0.0047
Total PUFA	28.36 ^a^	24.09 ^b^	17.47 ^c^	1.07	<0.0001
UFA/SFA	2.50 ^a^	2.26 ^b^	2.05 ^c^	0.06	0.0001
*n*-6	26.75 ^a^	22.66 ^b^	16.58 ^c^	1.00	<0.0001
*n*-3	1.61 ^a^	1.43 ^a^	0.89 ^b^	0.07	<0.0001
*n*-6/*n*-3	16.63 ^b^	16.00 ^b^	18.55 ^a^	0.26	<0.0001

CON, control diet; 25 and 50 HILM, HILM groups in which the soybean meal was replaced with 25 and 50% HILM, respectively; SEM, standard error of the means; SFA, saturated fatty acid; MUFA, monounsaturated fatty acid; PUFA, polyunsaturated fatty acid; UFA, unsaturated fatty acid; ^a,b,c^ Values with different superscripts in the same row are significantly different (*p* < 0.01).

**Table 6:** Effect of dietary *Hermetia illucens* larvae meal (HILM) inclusion level on the fatty acid profile in leg meat of broiler chickens.

Item	Dietary Treatment			SEM	*p*-Value
	CON	25 HILM	50 HILM		
Fatty acids					
C12:0 (Lauric)	0.63	0.89	0.88	0.16	0.470
C14:0 (Myristic)	0.67	0.77	0.77	0.06	0.463
C16:0 (Palmitic)	22.09	23.56	23.48	0.65	0.229
C18:0 (Stearic)	7.19	6.98	7.41	0.24	0.496
Total SFA	30.59	32.20	32.54	0.72	0.164
C16:1 *n*-7 (Palmitoleic)	4.75	5.30	5.33	0.41	0.549
C18:1 *n*-9 (Oleic)	36.23	36.35	36.68	0.67	0.904
C20:1 *n*-9 (Eicosenoic)	1.69	1.44	1.33	0.10	0.076
Total MUFA	42.67	43.09	43.35	0.97	0.895
C18:2 *n*-6 (Linoleic)	23.23	21.50	20.60	1.22	0.355
C18:3 *n*-6 (γ-Linolenic)	0.19	0.20	0.19	0.01	0.729
C18:3 *n*-3 (α-Linolenic)	0.27	0.26	0.31	0.01	0.081
C20:4 *n*-6 (Arachidonic)	0.73	0.70	0.75	0.12	0.951
C22:6 *n*-3 (Docosahexaenoic)	0.10	0.10	0.09	0.02	0.883
Total PUFA	24.52	22.73	21.94	1.32	0.413
UFA/SFA	2.22	2.05	2.03	0.07	0.121
*n*-6	24.15	22.40	21.54	1.30	0.404
*n*-3	0.37	0.36	0.39	0.28	0.726
*n*-6/*n*-3	64.86	63.49	60.40	4.07	0.772

CON, control diet; 25 and 50 HILM, HILM groups in which the soybean meal was replaced with 25 and 50% HILM, respectively; SEM, standard error of the means; SFA, saturated fatty acid; MUFA, monounsaturated fatty acid; PUFA, polyunsaturated fatty acid; UFA, unsaturated fatty acid.

**Table 7:** Essential elements and undesirable substances (mg/kg) in *Hermetia illucens* larvae meal (HILM).

Item	HILM	Permissible Limit (EC, 2002)
Essential elements		
Mg	5600	NA
S	562.24	NA
Essential trace elements		
Fe	560.90	NA
Cu	456.34	NA
Zn	142.15	NA
I	ND	NA
Cr	59.06	NA
Co	4.51	NA
Se	0.48	NA
Mn	101.08	NA
Undesirable substances		
Al	3.25	NA
F	0.01	150
As	0.01	2
Pb	0.01	10
Hg	<0.01	0.1
Cd	ND	2

EC, European Commission; NA, not applicable; ND, not detected.

**Table 8:** Essential elements and undesirable substances (mg/kg) in leg meat of broiler chickens fed *Hermetia illucens* larvae meal (HILM).

Item	Dietary Treatment			SEM	*p*-Value	Permissible Limit (EC, 2006)
	CON	25 HILM	50 HILM			
Essential elements						
Mg	233.60	232.74	222.45	4.24	0.191	NA
S	20.73 ^b^	25.19 ^b^	36.53 ^a^	1.80	<0.0001	NA
Essential trace elements						
Fe	7.72	8.27	9.29	0.43	0.070	NA
Cu	0.49	0.32	0.10	0.13	0.160	NA
Zn	11.65 ^b^	12.00 ^ab^	13.43 ^a^	0.44	0.035	NA
I	0.06	0.05	0.07	0.01	0.131	NA
Cr	<0.10	<0.10	<0.10	-	-	NA
Co	<0.10	<0.10	<0.10	-	-	NA
Se	<0.10	<0.10	<0.10	-	-	NA
Mn	<0.10	<0.10	<0.10	-	-	NA
Undesirable substances						
Al	6.48	4.45	2.62	1.65	0.319	NA
F	0.04	0.05	0.05	0.01	0.568	NA
As	<0.01	<0.01	<0.01	-	-	NA
Pb	<0.01	<0.01	<0.01	-	-	0.10
Hg	<0.01	<0.01	<0.01	-	-	NA
Cd	<0.01	<0.01	<0.01	-	-	0.05

CON, control diet; 25 and 50 HILM, HILM groups in which the soybean meal was replaced with 25 and 50% HILM, respectively; SEM, standard error of the means; EC, European Commission; NA, not applicable: ^a,b^ Values with different superscripts in the same row are significantly different (*p* < 0.05).

## Data Availability

The data presented in this study are available on reasonable request from the corresponding authors.
